# Navicular Disease*Paper read at a meeting of the Massachusetts Veterinary Association, February 24, 1892, W. Bryden, V. S.

**Published:** 1892-04

**Authors:** Williamson Bryden


### NAVICULAR DISEASE.*

By Williamson Bryden, V. S.

This article was intended for the Meeting of our Alumni Association, at Montreal, which was unavoidably postponed. With your indulgence, I will present to you a short contribution to one of the diseases peculiar to the locomotive organs of the horse, "*Navicular Disease.*"

Prof. Dick says: "By high authority it has been called *the curse upon all good horseflesh* (though in passing we remark it is rather the infliction of man than of any higher power)." He then asks, after fairly and generously quoting the opinions of Mr. Turner, Mr. Percival, and others, "Why, then, appear to differ when substantially we agree? Not that we are here arguing for concession which will compromise the truth; but we hold that the united, persevering ingenuity of scientific men has fully illustrated this disease; that it has predisposing causes, such as want of paring shoeing, and still more, bad shoeing, hereditary tendency of particular breeds, and high condition. . . . . In like manner it has manifested exciting causes, such as strain of the tendon and over-exertion, pressure of the sole, from travelling with a stone in the foot, etc., etc." The above are remarks by one who was a leader in our profession, and one whom all will long delight to honor and applaud. His efforts were directed to finding the "proximate cause" of this disease; consequently he endeavored to demonstrate that strain and over-exertion of the tendon, where it passes under the navicular bone, injures the "synovial capsule," between the tendon and navicular bone. This he believed to be the *primary* and *permanent* disease. Now, we do not quote the above because it is correct, but because it is as nearly being so as any thing on the subject found in works of much greater pretentions, published many years later. In both old and recent publications, we find confusing statements and accounts of the views and theories held by old veterinarians, many of them eminent in their day and generation, no doubt, and interesting too; but as picturesque figures in the history of the profession they are more conspicuous now, than for the exactness of their knowledge of those *diseases peculiar to the locomotive organs* of the horse, having failed to dis-

* Paper read at a meeting of the Massachusetts Veterinary Association, February 24, 1892, W. Bryden, V. S.

tinguish between such diseases and those liable to afflict all classes of animals, a fault still found with recent works, which repeat the same old story with slight variations, because their authors viewed the hoof from a standpoint that did not credit it with the far-reaching influences it possesses and exerts.

For this reason I trust you will not consider it over presumptuous on my part, with no better acquaintance with pathology than the ordinary practitioner possesses, to try to change the tale, be it ever so little. In the study of the subject, our attention is first directed to the fact that the soliped stands and moves on the points of his toes, which are covered and protected by a "horny box," a wonderful organism, having a limited elasticity and accommodation within it, and very liable to be affected adversely by surroundings and circumstances unfavorable to its organization and growth. This liability to change on the part of the hoof in its size, shape and quality, has a most important bearing on the organization, not merely of itself, but also of the structures contained within it, and on different parts of the limbs as well; indeed, the whole conformation of the animal is changed by acquired conditions of this wonderful "buffer," so unlike the foot of every other species of animal, that it must be the part *primarily affected*; the part that predisposes to, and determines the character of such diseases and the part that must be first treated, if the cause is to be philosophically studied, and successfully removed or overcome. For with an integument as soft and pliable as the skin, instead of the "horny box," it must be impossible to produce navicular disease, bone spavin, and many other diseases and defects with histories, such as theirs, which we are now familiar with; just as there must be many diseases peculiar to horses' limbs, that could not occur on the limbs of other species than solipeds. Let us now suppose a horse having received a severe blow with a club at the seat of ordinary bone spavin, or that his mate has kicked him there, and a bony growth or deformity results, somewhat similar, perhaps, to true bone spavin. It could hardly be regarded as good pathology to either name, or similarly classify diseases having such dissimilar histories and causes, any more than it would be to regard degeneration of the navicular bone, as met with in true navicular disease, as the same disease of the same bone, the result of inflammation from a nail having accidentally, or otherwise, penetrated it. In comparative work of this kind, clean lines must be drawn to give the student an exact, logical and intelligent appreciation of the subject. It must not be forgotten, that while the hoof is liable to be affected adversely by heat or cold, moisture or drouth, tear and wear, or idleness, etc., extrinsic and intrinsic influences; it is possible to guide its growth in a right or wrong direction, and it can be cultivated with as much success as the gardener acquires in guiding and training his bulbs and rare plants.

In practice we often meet with cases of navicular disease, and with cases of disease of the navicular bone, for they are quite distinct. For example, we find first, true navicular disease, a gradual contraction of the hoof from bad surroundings and neglect after birth, coupled possibly with some heredity, causing a slow change leading to *necrocis*, or gradual death of the bone. Second, a modified form of the above, hastened by such an accident as a prick with a nail that did or did not reach the bone. Third, disease of the navicular bone, from its having been, for example, injured by a nail or other instrument, the bone itself being penetrated; inflammation is set up *in the cancellated structures* of both the *interior of the bone* and the *compact structures* at the same time. Here then are three varieties at least:

In the first, Hereditary, or acquired predisposition.

In the second, Hereditary, or acquired predisposition, coupled with accident. In first and second then, we find contraction *both* as a cause and as a result or effect.

In the third, Accident only; here contraction may be present only as a *result* or *effect*.

To some, such fine distinctions may appear frivolous, but it must be evident that the history of a lame foot is of much importance, especially to the breeder; whether the derelict hoof is covering a case of true navicular disease, either of hereditary or of acquired origin, or a case of disease of the navicular bone, resulting from an accident or wound, and independent of the character of the hoof.

In looking over a recent edition of Prof. William's excellent Book of Surgery, one is surprised at statements like the following, on page 333, which must handicap the student in the study of such a subject. He says : "Contraction of the hoof is not a cause but an effect of disease ; an atrophy of the structures contained within the 'horny box' consequent upon diminished functional activity and adaptability of the hoof to the atrophied structures which it encloses and protects."

Continuing, he adds : " Prof. Dick said there was a kind of contraction of the hoof, in fact a *natural tendency* to this in the domesticated animal, arising from a want of moisture when he is confined in the stable. This kind of contraction, he (Prof. Dick)

maintained, did not cause lameness, as all the parts became adapted to the alteration of the hoof. Now in my (Prof. Williams') opinion, this kind of contraction would be the one most likely to cause lameness; indeed, it would be impossible for an animal not to be so, if the pressure of the drying hoof were sufficient to cause atrophy and absorption of the sensitive tissues within." Again, Prof. Williams asserts the following: "That horses' hoofs do become contracted, more especially at the heels, without lameness, I do not deny. I do not think, however, it is due to any want of moisture, but to the removal of the horn from the heels, etc., etc."

With all due deference to this eminent author and teacher, permit me to submit that the contention that "Contraction of the hoof is not a cause but an effect of disease" cannot be sustained, for it is quite possible of demonstration that it must be *both.* *Atrophy of the frog*, for example, when the result of mechanical pressure between the bars, is immediately arrested, and can be restored by paring out the bars and keeping them soft; in other cases the bars can be left strong, but the wall must be weakened so that it will bend outward with the same result. Sometimes it is an advantage to reduce the wall at the quarter, or quarters, and at the toe, from the coronet to the shoe. This prevents the hoof from collapsing, and when properly done, it relieves the circulation at the coronet, the hoof grows more robustly, the foot is less confined, and repair within it goes on more rapidly. But it is a most important matter to know how to pare a hoof, as it can be done right or wrong, too much, or too little, and it requires practice.

Contraction from "Atrophy of the structures contained within the 'horny box,' consequent upon diminished functional activity and adaptability" (Prof. Williams), and Prof. Dick's theory of *natural tendency of the hoof to contract*, is rather a fine drawn distinction, and quibbling likely to confuse many a student, for cases where both contentions are illustrated can easily be found every day. Then, why mix up the terms "Contraction" and "Navicular Disease?" "Contraction" is often found without "Navicular Disease," but "Navicular Disease" never without "Contraction," and within certain limits, always the same kind of "Contraction," too. One may claim that the hoof must be pared; the other, the opposite, and that adaptability is or is not "Contraction," might also be a contention, but can not get us out of old ruts. At the foot of page 335, Prof. Williams again informs us that "During the past five years, I (Prof. W.) have made numerous post-mortem examinations of "Navicular Disease," and am convinced that strain or laceration of the tendon is never a primary condition, and that the disease commences as an inflammation of the cancellated structures of the navicular bone, or of the cartilage upon its inferior surface." Impossible ; there is a history, and slow, gradual changes in the hoof and soft structures within it and covering the bone, without which it would not be "Navicular Disease."

Inflammation of the cancellated structures, and of the inferior part of the cartilage, can only be present as the result of an accident, never at the beginning of *true* Navicular Disease, which is a slow, gradual process, when not complicated ; the bone degeneration is always the result of *slow starvation* of the structures within the bone. The tissus surrounding the bone become disturbed, perhaps congested, by the pressure or interference of the hoof, especially the sole and bars. This extends to and affects the membrane covering the bone and lining the foramen, its functions are perverted, the elements intended to nourish the bones are refused admission or diverted ; a process of *Necrosis*, rather than *Caries*, gradually follows ; small particles of the rejected or arrested bone elements may then be transplanted or deposited on the exterior of the navicular bone, very much as in the degeneration of the cancellated structures in *true Bone Spavin.* It is said on page 342, that "Contraction of the foot always *succeeds* "Navicular Disease;" with equal truth it can be said that contraction of the hoof always *precedes* it. For it always does *both*, if the disease continues ; at first as an *exciting cause*, afterwards as a *concurrent affection or process.* For example : If a strong, robust, symmetrical, well-developed horse receives a serious injury, which compels the suspension of his limb for months (it may be free from much pain) it diminishes in size; every part being involved in the *general* atrophy, or wasting ; the hoof contracting as the inclosed tissues waste, from what Prof. Williams calls, "diminished functional activity and adaptability." The animal recovers without scar, pain, or lameness, the tissues protected by the elastic skin soon regain their volume, but the soft tissues protected by the contracted hoof cannot. The hoof, having shrunk, has acquired a shape and size determined mostly by the coffin bone within. When standing idle, there is *nothing to cause irritation*, but as soon as put to work, disturbances follow ; then instead of a general atrophy, we find atrophies and changes of particular parts, according to the shape, size, and quality of the hoof, the degree of disturbance, and the demand made on it.

*Changes*, the result of "diminished functional activity" and

"adaptability," and *changes*, the result of disturbance, especially at the extremities, are therefore quite different conditions.

The hoof peculiar to Navicular Disease may be a hind one, or a fore one ; usually small ; it has modifications. Consequently we find different degrees of the disease. One, or both heels are shortened ; one, or both heel cartilages are bent inwards; one, or both bars crowd ; one, or both halves of the frog diminish ; one, or both sides of the wall shrink, and the hoof is twisted, dry, and hard, toeing out or in. When long idle, it is often cold; but when exercised, it is hot. Every feature of the case proclaims mechanical interference by the "horny box," and demands treatment with the drawing knife, buttress, and rasp ; followed by persistent softening with poultices, or otherwise, which will encourage repair and growth, without the aid of the tortures now so universally popular in such cases.

Where we find such disturbance at the extremity, there must be strain of the tendon, for there is always *shortening* of it when there is atrophy ; just as we find *relaxation* in paralysis, when the injury is central. This is illustrated in cases of shortenings of the flexor tendons, as when the animal is compelled to walk only on the points of his toes (hoofs) ; in atrophy of the hip and tail muscles, the result of contraction of the heels and bone spavin, as when the tail is drawn to the *atrophied* side, instead of going to the side on which the muscles are strongest, as it does in paralysis, the result of a central lesion or an injury ; and again, in cases where the gaits and action *acquired* and *peculiar to* the horse are equivocal, or faulty, either from peripheral disturbance or inharmonious organization of their limbs, from this, or possibly some other cause.

The fact is, the hoof has never been appreciated at its proper value. No matter how defective it may have been, it has not been regarded as a factor of so much importance as it is, in diseases and wrong conditions of itself, which involve the limb above.

